# *Lactobacillus acidophilus* NCFM and *Lactiplantibacillus plantarum* Lp-115 inhibit *Helicobacter pylori* colonization and gastric inflammation in a murine model

**DOI:** 10.3389/fcimb.2023.1196084

**Published:** 2023-08-09

**Authors:** Siqi Shen, FeiFei Ren, Haiming Qin, Ihtisham Bukhari, Jing Yang, Dafang Gao, Arthur C. Ouwehand, Markus J. Lehtinen, Pengyuan Zheng, Yang Mi

**Affiliations:** ^1^ Henan Key Laboratory of Helicobacter pylori & Microbiota and Gastrointestinal Cancer, Marshall Medical Research Center, The Fifth Affiliated Hospital of Zhengzhou University, Zhengzhou, China; ^2^ Department of Gastroenterology, The Fifth Affiliated Hospital of Zhengzhou University, Zhengzhou, Henan, China; ^3^ R&D Health & Biosciences, Danisco (China) Holding Co. Ltd, Shanghai, China; ^4^ IFF Health & Biosciences, Global Health and Nutrition Science, Kantvik, Finland

**Keywords:** *Helicobacter pylori*, *Lactobacillus*, adhesion, inflammation, probiotic

## Abstract

**Purpose:**

To determine the role of Lactobacillus strains and their combinations in inhibiting the colonization of *H. pylori* and gastric mucosa inflammation.

**Methods:**

Human gastric adenocarcinoma AGS cells were incubated with H. pylori and six probiotic strains (*Lactobacillus acidophilus* NCFM, *L. acidophilus* La-14, *Lactiplantibacillus plantarum* Lp-115, *Lacticaseibacillus paracasei* Lpc-37, *Lacticaseibacillus rhamnosus* Lr-32, and *L. rhamnosus* GG) and the adhesion ability of *H. pylori* in different combinations was evaluated by fluorescence microscopy and urease activity assay. Male C57BL/6 mice were randomly divided into five groups (uninfected, *H. pylori*, *H. pylori*+NCFM, *H. pylori*+Lp-115, and *H. pylori*+NCFM+Lp-115) and treated with two lactobacilli strains (NCFM and Lp-115) for six weeks. *H. pylori* colonization and tissue inflammation statuses were determined by rapid urease test, Hematoxylin-Eosin (HE) staining, immunohistochemistry, and qRT-PCR and ELISA.

**Results:**

*L. acidophilus* NCFM, *L. acidophilus* La-14, *L. plantarum* Lp-115, *L. paracasei* Lpc-37, *L. rhamnosus* Lr-32, and *L. rhamnosus* GG reduced *H. pylori* adhesion and inflammation caused by *H. pylori* infection in AGS cells and mice. Among all probiotics *L. acidophilus* NCFM and *L. plantarum*, Lp-115 showed significant effects on the *H. pylori* eradication and reduction of inflammation *in-vitro* and *in-vivo*. Compared with the *H. pylori* infection group, the mRNA and protein expression levels of IL-8 and TNF-α in the six *Lactobacillus* intervention groups were significantly reduced. The changes in the urease activity (*ureA* and *ureB*) for 1-7h in each group showed that *L. acidophilus* NCFM, *L. acidophilus* La-14, *L. plantarum* Lp-115, and *L. rhamnosus* GG effectively reduced the colonization of *H. pylori*. We observed a higher ratio of lymphocyte and plasma cell infiltration into the lamina propria of the gastric mucosa and neutrophil infiltration in *H. pylori*+NCFM+Lp-115 mice. The infiltration of inflammatory cells in lamina propria of the gastric mucosa was reduced in the *H. pylori*+NCFM+Lp-115 group. Additionally, the expression of IFN-γ was decreased significantly in the NCFM and Lp-115 treated C57BL/6 mice.

**Conclusions:**

*L. acidophilus* NCFM and *L. plantarum* Lp-115 can reduce the adhesion of *H. pylori* and inhibit the gastric inflammatory response caused by *H. pylori* infection.

## 1 Introduction

*Helicobacter pylori* infect over 50% of the population worldwide, and the World Health Organization (WHO) has listed *H. pylori* as a class I carcinogen since 1994 (Hooi et al., 2017; Shah et al., 2021). *H. pylori* infection is closely related to the occurrence and development of various gastrointestinal diseases, such as chronic gastritis, peptic ulcer, gastric cancer, and gastric mucosa associated lymphoid tissue lymphoma (Sugano et al., 2015; Liu et al., 2018; Liou et al., 2020; Robinson and Atherton, 2021). Currently, the primary method to eradicate *H. pylori* is a quadruple therapy based on a proton pump inhibitor, two antibiotics, and a bismuth agent (Fallone et al., 2016; Chey et al., 2017). However, the antibiotic resistance rate of *H. pylori* has increased, and the side effects of the eradication therapy can be severe (Savoldi et al., 2018). Therefore, searching for novel and efficient *H. pylori* management options has become an urgent aim (Fallone et al., 2019).

Studies on probiotics and *H. pylori* have made significant progress recently, thus, increasingly being used in routine clinics (Suez et al., 2019; Sousa et al., 2022). Currently, blends of probiotics are the most widely studied, but little is known about the antagonistic or synergistic effects of the different probiotic strains (Vieira et al., 2013; Ouwehand et al., 2018; Simon et al., 2021). To manage *H. pylori* infection, the Maastricht VI/Florence Consensus Report mentioned that only some probiotics could effectively reduce gastrointestinal side effects in *H. pylori* eradication therapy, suggesting strain-specific efficacy (Malfertheiner et al., 2022). However, the European Society of Paediatric Gastroenterology and Hepatology later updated the guidelines. They considered that the existing evidence was insufficient to support the routine use of single or compound probiotic strains in treating *H. pylori* to reduce adverse reactions and improve the eradication rate (Jones et al., 2017). Therefore, probiotics are mainly used as an adjunct to *H. pylori* eradication therapy, and only a few reports are available for using probiotics as a single treatment for *H. pylori* infection, and further investigations are warranted.

The applications of certain probiotics, such as lactobacilli, fecal bacteria, *Bifidobacterium* spp., *Saccharomyces* spp., and *Bacillus licheniformis*, to assist in *H. pylori* eradication have been incorporated into *H. pylori* treatment guidelines (Shi et al., 2019). These probiotics attenuate the gastrointestinal adverse effects of *H. pylori* eradication therapy, but whether they can improve *H. pylori* eradication rates is controversial (Liu et al., 2018). Meta-analyses on the efficacy of multiple probiotic strains in treating *H. pylori* have shown the most significant effects with lactobacilli (Lu et al., 2016; McFarland et al., 2016). In related studies of using lactobacilli for treating *H. pylori* infection, certain lactobacilli such as *Lactobacillus acidophilus*, *Lacticaseibacillus rhamnosus*, *Lactiplantibacillus plantarum*, *Lacticaseibacillus paracasei*, *Limosilactobacillus reuteri* and *Lactobacillus delbrueckii* subsp. *bulgaricus* can effectively manage *H. pylori* infection (Zhao et al., 2018; Chen et al., 2019; Yoon et al., 2019; Asgari et al., 2020; Lin et al., 2020; Dargenio et al., 2021) but underlying mechanisms are not well explained. However, it has been speculated that lactobacilli interfere with the adhesion of *H. pylori* to the mucosa and down-regulate the immune and inflammatory mediators (Keikha and Karbalaei, 2021).

In this study, six lactobacilli strains with good acid, bile salt, and digestive enzyme resistance, combined with good mucosal adhesion were used in screening experiments to identify probiotics that inhibit the adhesion and inflammatory response to *H. pylori*. We tested the selected probiotics in the *H. pylori* infected AGS cell line and mouse models. The results of the cell model experiments provided a basis for probiotic strain selection for the eradication of *H. pylori* in the mouse model.

## 2 Materials and methods

### 2.1 Bacterial strains, cell lines and animals

*H. pylori* P12 and *H. pylori* P12-GFP strains were provided by the Max Planck Institute for Infection Biology and *H. pylori* SS1 (ATCC 43504) (Lee et al., 1997) was provided by the University of Western Australia (UWA), Australia. *H. pylori* strains were cultured on Columbia agar containing 7% sterile defibrinated sheep blood (Bianzhen, Nanjing, China), 20μg/ml vancomycin (Meilunbio, Dalian, China), 10μg/ml polymyxin (Meilunbio), 10μg/ml amphotericin B (Meilunbio), 10μg/ml trimethoprim (Sigma, St. Louis, USA), then placed in an incubator containing 5% O_2_ and 10% CO_2_, cultured at 37°C, subcultured once every three days, and used for the experiment after subculture. The tested lactobacilli were provided by Danisco China (Shanghai, China): *Lactobacillus acidophilus* NCFM (ATCC 7003969), *L. acidophilus* La-14 (ATCC SD5212), *Lactiplantibacillus plantarum* Lp-115 (ATCC SD5209), *Lacticaseibacillus paracasei* Lpc-37 (ATCC SD5275), *Lacticaseibacillus rhamnosus* Lr-32 (ATCC SD5217) and *L. rhamnosus* GG (ATCC 53103). After the Gram Staining Kit (Solarbio, Beijing, China) was used to identify the bacterial morphology, the lactobacilli were cultured anaerobically in MRS broth (Solarbio) at 37 °C 48 hours and then subcultured (**Figure S1**).

The human gastric adenocarcinoma cell line (AGS) was purchased from the Institute of Biochemistry and Cell Biology of the Chinese Academy of Sciences (Shanghai, China) and cultured in RPMI 1640 medium (Thermo Fisher, Waltham, MA, USA) supplemented with 10% fetal bovine serum at 37°C and 5% CO_2_ in a humidified incubator.

Fifty male C57BL/6 mice, Specific Pathogen Free (SPF), four weeks old, were purchased from Zhejiang Vital River Laboratory Animal Technology Co. Ltd. (Zhejiang, China). All the animals were housed under standard conditions (SPF grade animal room with individually ventilated cages; temperature range from 23°C to 25°C, humidity range from 50% to 60%, 12/12 hours light/dark cycle, food and water were provided ad libitum). The experimental steps and ethics were approved by the ethics committee of the Fifth Affiliated Hospital of Zhengzhou University (KY2022002).

### 2.2 Cytokine profiles quantification by ELISA

After infection, the culture medium was collected by centrifugation at 12000x rpm for 5 min to remove cell debris and bacteria and collect the supernatant. The concentration of interleukin (IL)-8 and tumor necrosis factor (TNF)-α were detected by human IL-8 ELISA Kit (Elabscience, Wuhan, China) and human TNF-α ELISA Kit (Elabscience, Houston, TX, USA) respectively, by following the guidelines of the manufacturer. All experiments were performed in triplicate.

### 2.3 *H. pylori* and lactobacilli co-infection model *in vitro*

*H. pylori* P12 and lactobacilli were cultured overnight in BHI (Thermo Fisher) and MRS broth. After centrifugation at 5000 x rpm for 8 min and 4000 x rpm for 5 min, the supernatant was discarded, and bacteria were harvested and resuspended in 1 ml serum-free RPMI 1640 medium.

Overnight cultured AGS cells were co-incubated with lactobacilli (multiplicity of infection (MOI) = 100) and *H. pylori* P12 (MOI = 100) for 6h. After the incubation period, the supernatant was harvested for ELISA and cells were harvested for RNA isolation.

### 2.4 Adhesion of *H. pylori* on AGS cell

AGS cells were cultured in two 12 well plates and incubated with lactobacilli (MOI = 100) and *H. pylori* P12-GFP (MOI = 100) for 6h. At the end of incubation, one plate of the cells was washed thrice with PBS and photographed by fluorescence and white light. A urease detection reagent was prepared by adding concentrated hydrochloric acid into the PBS (pH=7.4) to adjust the solution to pH=6.8. Urea (Solarbio) and phenol red (Solarbio) were weighed and added to reach concentrations of 110mmol/L and 10mg/L, respectively, and then dissolved by vigorously shaking. To the other 12well plates, 1 ml urease detection reagent was added. After 1-7 hours of reaction, 80μl medium was withdrawn to record the absorbance value at 540nm(Shmuely et al., 2004; Rokka et al., 2008; Tharmalingam et al., 2014; Yang et al., 2020).

### 2.5 Quantitative reverse transcription PCR (qRT-PCR)

AGS cells and mouse gastric mucosal tissue were lysed with RNAiso plus (TaKaRa, Kyoto, Japan), and gastric mucosal tissue needed to be assisted by an ultrasonic crusher. According to the manufacturer’s instructions, cDNA was converted using the ReverTra Ace qPCR RT Kit (TOYOBO, Shanghai, China). qRT-PCR was performed using 2×ChamQ Universal SYBR qPCR Master Mix (Vazyme, Nanjing, China) in a Roche Lightcycler480II system based on the manufacturer’s recommendations. Primer sequences were designed in NCBI Primer-BLAST (**Table 1**). The relative gene expression was determined using the 2^-ΔΔCt^ method. All experiments were repeated thrice.

**Table 1:** Primers for the quantification of inflammatory factors in AGS cells and mouse gastric tissue.

Species	Gene	Primer	Nucleotide Sequence(5’-3’)
Human	*TNF*	Forward	CCCAGGGACCTCTCTCTAATCA
	*TNF*	Reverse	GCTACAGGCTTGTCACTCGG
	*Cxcl8*	Forward	ACTGAGAGTGATTGAGAGTGGAC
	*Cxcl8*	Reverse	AACCCTCTGCACCCAGTTTTC
	*GAPDH*	Forward	GGTATCGTGGAAGGACTCATGAC
	*GAPDH*	Reverse	ATGCCAGTGAGCTTCCCGTTCAG
Mouse	*ureA*	Forward	GCTGGTGCGATTGGCTTTA
	*ureA*	Reverse	GGATAGCGACTTGCACATCGT
	*ureB*	Forward	GCCCACTTCTACAGAACCGACATAC
	*ureB*	Reverse	AGGCGATAACGACAACTTCAGGATC
	*Il10*	Forward	CCAGGGAGATCCTTTGATGA
	*Il10*	Reverse	AACTGGCCACAGTTTTCAGG
	*Il4*	Forward	GGTCTCAACCCCCAGCTAGT
	*Il4*	Reverse	GCCGATGATCTCTCTCAAGTGAT
	*Ifng*	Forward	CAGGCCATCAGCAACAACATAAGC
	*Ifng*	Reverse	AGCTGGTGGACCACTCGGATG
	*Cxcl15*	Forward	CGGCAATGAAGCTTCTGTAT
	*Cxcl15*	Reverse	CCTTGAAACTCTTTGCCTCA
	*GAPDH*	Forward	GCTGAGTATGTCGTGGAG
	*GAPDH*	Reverse	TCTTCTGAGTGGCAGTGAT

### 2.6 Establishing the model of *H. pylori* infection and lactobacilli intervention in mice

**Figure 1:**
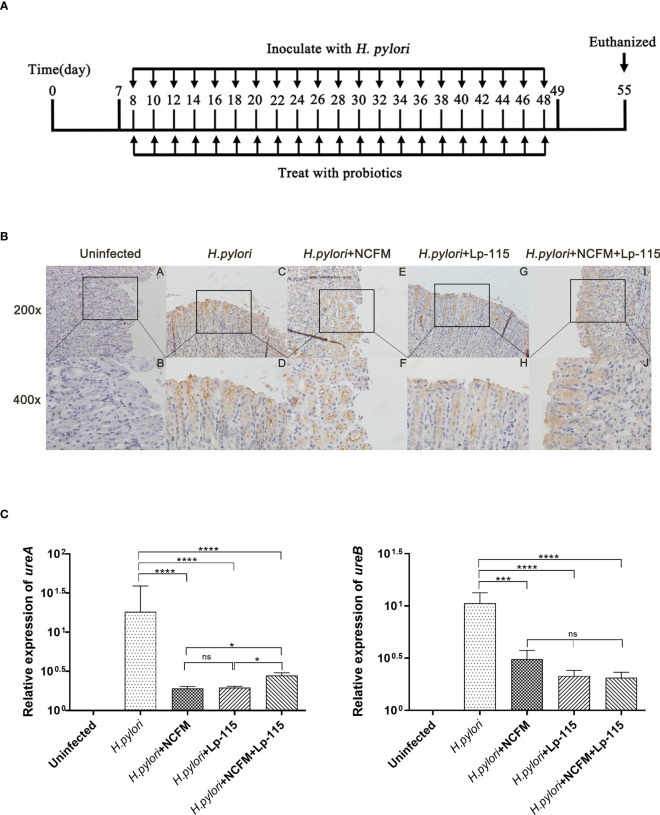
*Lactobacillus acidophilus* NCFM and *Lactiplantibacillus plantarum* Lp-115 suppress *Helicobacter pylori* adhesion in mice. **(A)** Mice were fed with *H*. *pylori* SS1 only or with *L. acidophilus* NCFM and/or *L. plantarum* Lp-115 for 6 weeks. **(B)** The colonization of *H*. *pylori* was identified by immune histochemistry. Uninfected group; *H*. *pylori* group; *H. pylori*+ NCFM group; *H. pylori* + Lp-115 group; *H. pylori* +NCFM+Lp-115 group. **(C)** The colonization of *H. pylori* was identified by the expression of *ureA* and *ureB* by qRT-PCR. Mice were coinfected with *H*. *pylori* SS1 and *L. acidophilus* NCFM and/or *L. plantarum* Lp-115. The mRNA levels of *ureA* and *ureB* were determined as described. Each experiment result shows the mean ± standard deviation of three independent experiments. * (*P*< 0.05); **(*P*<0.01); ***(*P*<0.001); ns, not significant.

SPF C57BL/6 mice (male 4 weeks old) were obtained from Zhejiang Vital River Laboratory Animal Technology Co., Ltd. (Zhejiang, China) and fed on Laboratory Rodent Diet 5001. The mice were randomly divided into 5 groups (Uninfected, *H. pylori* SS1, *H. pylori* SS1+*L. acidophilus* NCFM, *H. pylori* SS1+*L. plantarum* Lp-115, *H. pylori* SS1+*L. acidophilus* NCFM and *L. plantarum* Lp-115) of 10 individuals each after one week of adaptation. The control group was gavaged with PBS, and the experimental groups were gavaged with *H. pylori* SS1 (1*10^9^ CFU, 0.2ml/piece) only or combined with the corresponding lactobacilli (1*10^9^ CFU, 0.2ml/piece) (1:1) or together (1:1:1) once every other day for 6 weeks (**Figure 1A**).

### 2.7 Rapid urease test (RUT)

Mouse gastric mucosa was analyzed for urease activity using the rapid urease Kit (Sanqiang, Fujian, China) as instructed by the manufacturer. The color change was determined, when the solution turns pink or red, the urease test is positive; when it remains yellow, the urease test is negative.

### 2.8 Hematoxylin-eosin (HE) staining and immunohistochemistry (IHC)

Gastric tissues were fixed with 4% paraformaldehyde, embedded in paraffin, cut into 4mm sections, and stained by HE and immunohistochemical methods. The Chronic gastritis histological grade scale was used to evaluate the samples (Fang et al., 2018). Five histological changes were graded: *H. pylori*, chronic inflammation, activity, atrophy, and intestinal metaplasia. Each histological change was classified into one of four grades: none, mild, moderate, and severe. According to the new Sydney system, the degree of inflammation and lymphocyte infiltration of gastric tissue after *H. pylori* infection were evaluated (Kim et al., 2020; Maluf et al., 2020). Immunohistochemistry for *H. pylori* was performed using a Rabbit anti-*H. pylori* polyclonal antibody (Cell Marque) (ZSGB, Beijing, China), and the colonies of *H. pylori* in mouse gastric mucosa were identified.

### 2.9 Statistics

All statistical analyses were performed using GraphPad Prism 9 software. During the processing of experimental data, the values whose deviation from the average value of the same group of data exceeded three times the standard deviation were considered outliers and eliminated. We performed one-way ANOVA on raw and lg-converted data to compare the multi groups. The Bonferroni and Tukey tests were conducted to calculate the statistical significance among the groups. P-value < 0.05 was considered significant for all statistical analyses.

## 3 Results

### 3.1 Six lactobacilli strains inhibit the adhesion of *H. pylori* with AGS cells

**Figure 2:**
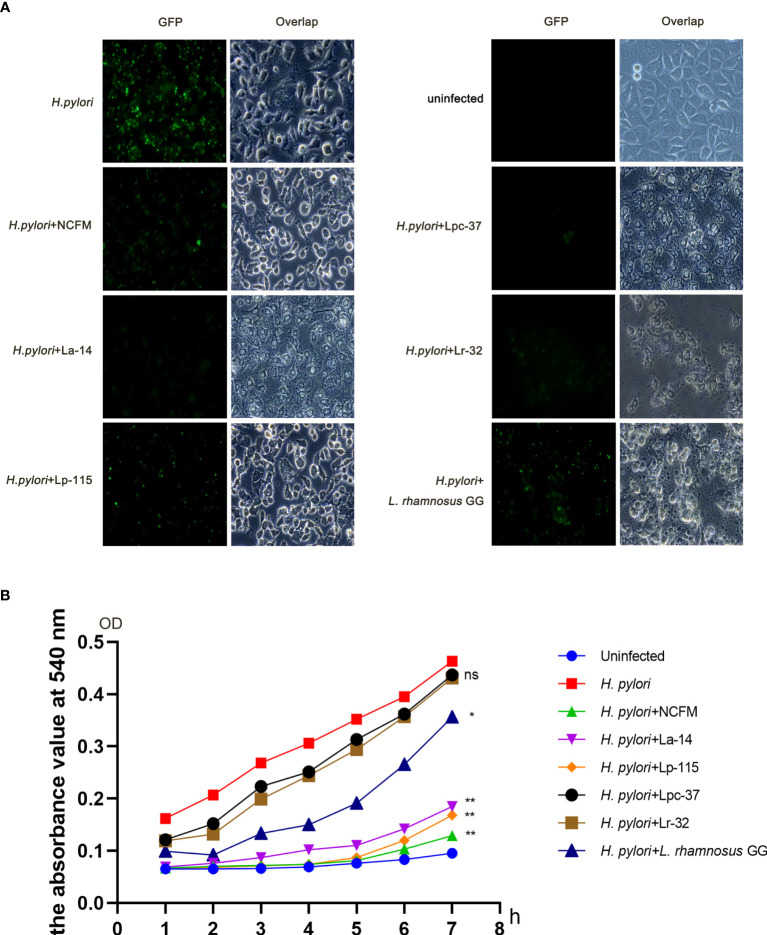
Six lactobacilli strains inhibit the adherence of *Helicobacter pylori* to AGS cells. **(A)** Fluorescence microscope images showing the colonization of *H*. *pylori* P12-GFP on AGS cells alone or upon intervention with six lactobacilli: *Lactobacillus acidophilus* NCFM, *L. acidophilus* La-14, *Lactiplantibacillus plantarum* Lp-115, *Lacticaseibacillus paracasei* Lpc-37, *Lacticaseibacillus rhamnosus* Lr-32 and *L. rhamnosus* GG). **(B)** Urease activity assay shows interference with the colonization of *H*. *pylori* P12-GFP on AGS cells in 1-7 hours by the six tested probiotic strains. *(*P*<0.05); **(*P*<0.01), compared to *H*. *pylori*. ns, not significant.

To compare the effect of the six selected lactobacilli interfering with *H. pylori* adhesion, AGS cells were co-infected with *H. pylori* P12-GFP and the six lactobacilli strains (MOI=100) respectively for 6 hours. After infection, cells and GFP-positive *H. pylori* were measured under fluorescence microscopy. Upon comparative observation under fluoroscopy, the amount of GFP-positive *H. pylori* in the lactobacilli groups was less than in the *H. pylori* only infected group. Furthermore, the morphology of AGS cells was relatively normal in the *L. acidophilus* NCFM and *L. plantarum* Lp-115 treated cells compared with the other lactobacilli treated cells, indicating less cell stress on the AGS cells compared with other lactobacilli strains (**Figure 2A**).

To further quantify the inhibition effect between different lactobacilli, we used a urease activity assay for the co-infection plate and recorded the absorbance at 540nm. The absorbance values of the six intervention groups were decreased to different degrees from 1 to 7 hours compared with that of the *H. pylori* infected group. For further quantification analysis, the absorbance values at each group’s 7h time point were taken into a bar chart for statistical analysis. The absorbance values of the seventh hour were statistically analyzed. The data suggest that samples of *L. acidophilus* NCFM, *L. acidophilus* La-14, *L. plantarum* Lp-115 and *L. rhamnosus* GG had significant differences compared with samples of *H. pylori* (*P*<0.05), indicating that these four lactobacilli strains can effectively reduce the colonization of *H. pylori* (**Figure 2B**).

### 3.2 Six probiotic strains inhibit *H. pylori* induced inflammation in AGS cells

**Figure 3:**
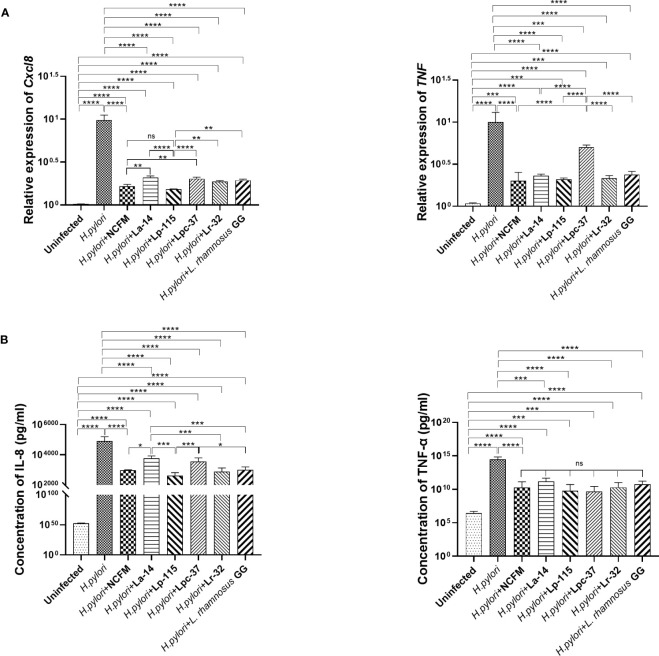
Inhibitory effects of the six probiotic strains on *Helicobacter pylori*-induced inflammation in AGS cell line. AGS cells were co-infected with the probiotic strains: *Lactobacillus acidophilus* NCFM, *L. acidophilus* La-14, *Lactiplantibacillus plantarum* Lp-115, *Lacticaseibacillus paracasei* Lpc-37, *Lacticaseibacillus rhamnosus* Lr-32 and *L. rhamnosus* GG and *H*. *pylori* P12 at a multiplicity of infection (MOI) 100 for 6 hours. **(A)** The mRNA levels of Cxcl8 and TNF the cells and **(B)** the protein concentrations of IL-8 and TNF-α in the supernatant. The results of each experiment are shown as mean ± standard deviation of three independent experiments. *(*P*<0.05); **(*P*<0.01); ***(*P<*0.001); ****p<0.0001; ns, not significant.

To compare the inhibitory effect between the six probiotic strains, we performed a co-infection model of *H. pylori* and the strains (MOI=100) in AGS cell culture for 6 hours. We analyzed the mRNA and protein levels of IL-8 (Cxcl8) and TNF-α (TNF) to determine whether these probiotic strains transcriptionally regulate the inflammatory markers. The expression of these markers is commonly altered upon *H. pylori* infection. Expression of *Cxcl8* and *TNF* mRNA with all six probiotic strains was significantly reduced compared with the *H. pylori* infected group (*P*<0.001). In *Cxcl8*, Lp-115 showed significant difference than other probiotics except NCFM, while pattern of NCFM was significantly lower than La-14 and Lpc-37 (*P*<0.01). The comparison of other probiotics including La-14, Lpc-37, Lr32 and GG did not show any significant difference (*P>*0.01). In *TNF*, only Lpc-37 was found to have significant difference than other probiotics in the group (*P*<0.01) (**Figure 3A**).

Consistent with the mRNA results, IL-8 and TNF-α in the cell supernatant were significantly decreased in the probiotic treatments compared with the *H. pylori-*infected group (*P*<0.001). In IL-8, NCFM was significantly lower than La-14, while Lp-115 showed significant difference than the La-14 and Lpc-37 (*P*<0.01). In TNF-a, the inhibitory effect of the six selected probiotic strains did not show significant differences (**Figure 3B**). Combined with the anti-adhesion results, *L. acidophilus* NCFM and *L. plantarum* Lp-115 had a good effect and less cell stress. Therefore, *L. acidophilus* NCFM and *L. plantarum* Lp-115 were further selected for validation in the *H. pylori* infected mouse model.

### 3.3 *L. acidophilus* NCFM and *L. plantarum* Lp-115 suppress *H. pylori* colonization in mice

To further validate whether *L. acidophilus* NCFM and *L. plantarum* Lp-115 alone or in combination can counteract *H. pylori* colonization and attenuate gastric inflammation *in vivo*, C57BL/6 mice were infected with *H. pylori* SS1 and co-administered *L. acidophilus* NCFM and *L. plantarum* Lp-115 with for 6 weeks (**Figure 1A**). After co-administration the mice were euthanized, and the gastric tissues were assessed for *H. pylori* infection by the rapid urease test (**Figure S2**). The *H. pylori* adhesion on gastric tissues was then analyzed by immunohistochemistry (IHC) assays (**Figure 1B**), which showed that *L. acidophilus* NCFM and/or *L. plantarum* Lp-115 intervention groups had comparably less *H. pylori* adhesion than the *H. pylori* infected group. To further validate the *H. pylori* colonization in different groups, the mRNA expression of *ureA* and *ureB* was tested from gastric tissues of all groups (**Figure 1C**), which showed that *L. acidophilus* NCFM and/or *L. plantarum* Lp-115 intervention groups had less expression of *ureA* and *ureB* compared with *H. pylori* infected group (*P*<0.001). These results show that *L. acidophilus* NCFM and *L. plantarum* Lp-115 alone or combined can reduce *H. pylori* colonization on gastric mucosa in mice.

### 3.4 *L. acidophilus* NCFM and *L*. *plantarum* Lp-115 suppress *H. pylori* induced inflammation in mice

**Figure 4:**
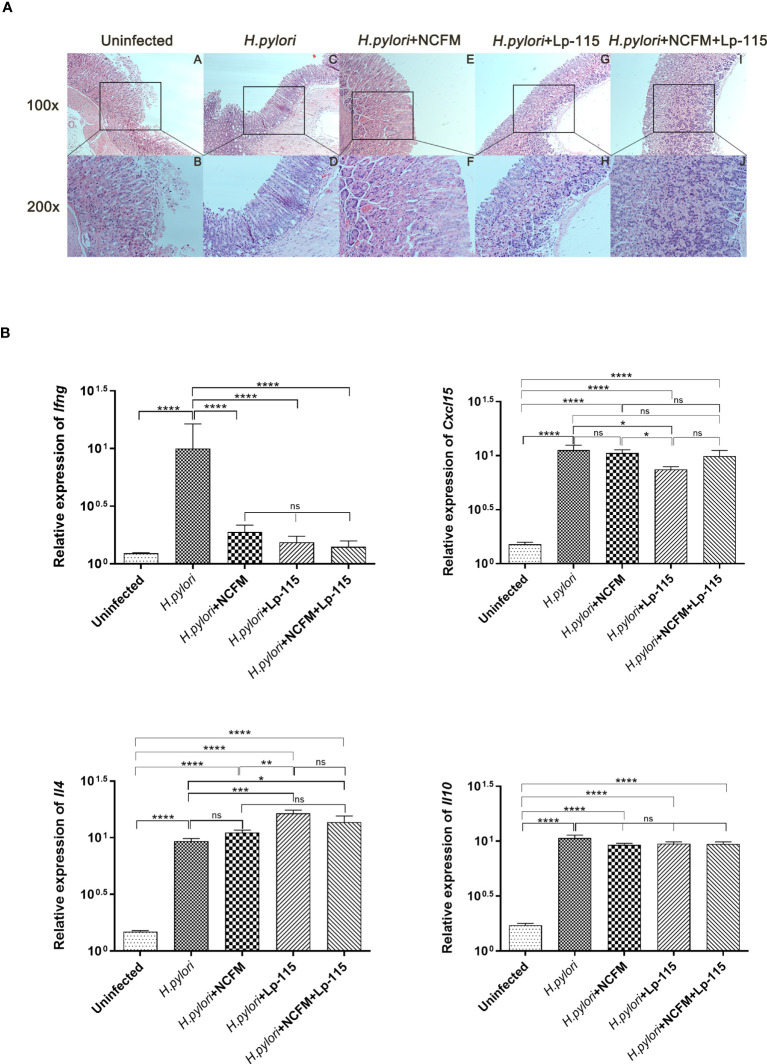
*Lactobacillus acidophilus* NCFM and *Lactiplantibacillus plantarum* Lp-115 suppress *Helicobacter pylori* inflammation in mice. **(A)** The inflammation of *H. pylori* was identified by HE. Uninfected group; *H*. *pylori* group; *H. pylori*+ NCFM group; *H*. *pylori* + Lp-115 group; *H. pylori* +NCFM+Lp-115 group. **(B)** The mRNA expression level of *Il4, Cxcl15, Il10, and Ifng* in each group. ns(*P*≥0.05); *(*P*<0.05); **(*P*<0.01); ***(*P*<0.001); ****(*P*<0.0001); ns, not significant.

To further investigate whether *L. acidophilus* NCFM and *L. plantarum* Lp-115 can attenuate *H. pylori* colonization and gastric inflammation *in vivo*, gastric tissues from the five study groups were analyzed by Hematoxylin-Eosin staining (HE stain). Compared with the uninfected group, the gastric lamina propria of mice in the *H. pylori* infected group showed more lymphocyte, plasma cell, and neutrophil infiltration in the active phase. Incidentally, the mice also had local thinning of the mucosal layer, reduction of the glands propria, and thickening of the muscularis mucosae (**Figure 4A**). Compared with the *H. pylori* group, the inflammatory cells infiltrating the lamina propria of the gastric mucosa in the *H. pylori* + NCFM group, *H. pylori* + Lp-115 group, and *H. pylori*+NCFM+Lp-115 group were reduced to different degrees, which indicated that *L. acidophilus* NCFM and *L. plantarum* Lp-115 could ameliorate the *H. pylori* induced gastric inflammation (**Figure 4A**).

To further verify if NCFM and/or Lp-115 can inhibit *H. pylori* induced Th1 type inflammation, the mRNA expression of *Il4* (IL-4), *Cxcl15* (CXCL15), *Il10* (IL-10), and *Ifng* (IFN-γ) was measured by qRT-PCR from mouse gastric mucosal tissue. The results showed that NCFM and Lp-115 reduced the expression of *Ifng* and promoted the expression of *Il4* induced by *H. pylori* in C57BL/6 mice. In *Il4* expression, the difference between the Lp-115 and H. pylori groups was the most significant (*P*<0.001). In *Cxcl15*, only the Lp-115 group was significantly different from the *H. pylori* group (*P*<0.01); therefore, the overall anti-inflammatory effect of Lp-115 was more pronounced than that of NCFM. The expression of *Cxcl15* in the group receiving the combination of Lp-115 and NCFM was not significantly different from that in the *H. pylori* group. The expression of *Il4* was lower than that in the Lp-115 group (**Figure 4B**), indicating that the two LAB strains had no compound effect in improving inflammation caused by *H. pylori* infection. In conclusion, NCFM and/or Lp-115 can reduce *H. pylori* induced Th1 type inflammation (*Ifng* expression) in C57BL/6 mice and tend to transform Th2 type inflammation (*Il4* expression).

## 4 Discussion

Currently, quadruple therapy is the standard treatment for *H. pylori* eradication, but it has drawbacks. There is an increasing incidence of drug resistance and the misuse of antibiotics for *H. pylori* eradication can cause gastrointestinal disorders, gastrointestinal microbiota dysbiosis and other adverse effects (Hu et al., 2017). Modulation of the gastrointestinal microecology by microbial agents could represent a novel therapy or adjunct therapy for the current quadruple treatment. Furthermore, previous studies have shown that probiotics can modulate immune function, balance normal gastrointestinal microbiota, and reduce the side effects of antibiotics (Lu et al., 2016; Wang et al., 2017; Goderska et al., 2018), but may also inhibit *H. pylori* adhesion and gastric inflammation, suggesting beneficial effects. Although probiotics have advantages in aiding the eradication of *H. pylori* infection during conventional therapy, potential risks still exist. For some immunocompromised people, some strains of lactobacilli under certain rare conditions can cause infections (Liong, 2008). Therefore, selecting safe and suitable probiotic strains to support H. pylori eradication and management therapy is necessary.

Previous studies have shown that selected strains of lactobacilli can inhibit adherence of *H. pylori* to the gastric mucosa (Song et al., 2019; Zuo et al., 2019). In our study, we screened six probiotic strains and found that *L. acidophilus* NCFM and/or *L. plantarum* Lp-115 can inhibit *H. pylori* adhesion in an *in vitro* AGS cell line model and *in vivo* gastric mucosa of C57/BL6 mice, indicating preclinical evidence of these two strains for potential clinical use. However, the mechanisms by which NCFM and Lp-115 inhibit *H. pylori* colonization remains to be explored. Some studies have reported that probiotics’ effects are strain specific in inhibiting the colonization of *H. pylori*. For example, some *Lactobacillus* spp. such as *L. acidophilus* and *L. bulgaricus* have a high affinity for gastric epithelial cells, and they can protect the gastric mucosa by blocking or inhibiting the adhesion of *H. pylori* to gastric epithelial cells (de Klerk et al., 2016; Takeda et al., 2017; Zhao et al., 2018; Song et al., 2019). Some lactobacilli, such as *L. plantarum* and *Ligilactobacillus salivarius*, cannot compete with *H. pylori* for the binding site on the gastric mucosa but inhibit the activity of *H. pylori* through the antibacterial properties of metabolites, including Lactic acid and hydrogen peroxide (de Klerk et al., 2016; Takeda et al., 2017; Zhao et al., 2018; Song et al., 2019). Therefore, the immune system’s independent effects of probiotics against *H. pylori* infection may include affecting *H. pylori* gastric adhesive colonization or inhibiting *H. pylori* activity through the bacteriostatic properties of metabolites. These effects and other specific mechanisms need to be explored further.

*H. pylori* infection induces inflammation of gastric mucosa and expression of cytokines such as TNF-α or chemokines like CXCL8 (also known as IL-8) (Panpetch et al., 2016; Zhao et al., 2020; Tang et al., 2021). Previous studies have shown that *L. rhamnosus* GMNL-74 and *L. acidophilus* GMNL-185 reduce *H. pylori* induced gastric inflammation (Chen et al., 2019; Song et al., 2019). In the current study, we are showing for the first that *L. acidophilus* NCFM and/or *L. plantarum* Lp-115 inhibit *H. pylori* P12 induced IL-8 and TNF-α expression *in vitro*, which is consistent with previous studies analyzed other *L. acidophilus* strains (Ryan et al., 2009; Hwang et al., 2012). Previous studies have shown that *H. pylori* SS1 can induce T-helper cell type 1 (Th1) driven inflammation in C57BL/6 mice and that lactobacilli can suppress this response and may thus be involved in modulating Th1/Th2 balance (Boltin, 2016; Asgari et al., 2018; Asgari et al., 2020). In the *H. pylori* SS1 infected C57BL/6 mouse model, our study also confirmed that *L. acidophilus* NCFM and/or *L. plantarum* Lp-115 could inhibit *Ifng* but increase *Il4* expression, consistent with the previous report that *L. acidophilus* can turn *H. pylori* induced Th1 type inflammation into Th2 type inflammation in C57BL/6mice(Boltin, 2016). Helper T cells (Th1, Th2) are essential factors in immunity and the main effector molecules of Th1 are IFN-γ and IL-12, but IL-8 (CXCL15 in mice) may also contribute to the inflammation. The primary effector molecule of Th2 mediated inflammation is IL-4, whereas IL-10 may contribute to inhibiting Th2 responses. The two kinds of helper T-cells regulate and inhibit each other by secreting different factors to maintain the balance of the Th1 and Th2 (Schmitt and Ueno, 2015; Jafarzadeh et al., 2018; Saravia et al., 2019). Previous studies demonstrated that lactobacilli strains could balance the Th1/Th2 immune response. Certain *L. plantarum* strains can maintain normal intestinal immune function by stimulating the secretion of cytokines and regulating the Th1/Th2 balance (Xie et al., 2015; Boltin, 2016; Meng et al., 2019). Conversely, one study showed that *L. rhamnosus* GG could increase the number of CD4^+^ T lymphocytes, assist in differentiating Th cells and enhance Th1 immune responses (Shi et al., 2020). In this study, the intervention of *L. acidophilus* NCFM and *L. plantarum* Lp-115 might alleviate *H. pylori* infection induced host inflammatory response by down regulating local Th1 immune response in the gastric mucosa (inhibiting proinflammatory factor IFN-γ) while promoting Th2 response to produce the anti-Th1 cytokine IL-4. Thus, *L. acidophilus* NCFM and *L. plantarum* Lp-115 may play an essential role in promoting the differentiation of T cells into Th2 cells to balance *H. pylori* induced Th1 inflammation.

Although the focus of the study was not safety related, it shows that while the tested strains have different efficacy, they are not negatively affecting the AGS cell line or the *H. pylori* infected mice, which is in line with earlier reports (Daniel et al., 2006; Morovic et al., 2017). Thus, their choice of probiotic strain and rational application must be seriously considered. Based on the classification of risk factors posed by individuals, the safest, most effective, and most affordable lactobacilli to manage *H. pylori* infection should be selected for further investigation.

In conclusion, probiotic health benefits are strain-specific; thus, data specific for strain and health benefits should be investigated. After screening several strains, we chose two safe lactobacilli candidate strains: *L. acidophilus* NCFM and *L. plantarum* Lp-115, which inhibit *H. pylori* adhesion and host inflammatory responses in cell line and mouse models. *H. pylori* has a high infection rate and high prevalence of drug resistance worldwide. The current study presented a unique value in managing *H. pylori in vitro* and *in vivo*. The clinical intervention study with the two probiotic strains or their combination is warranted.

## Data availability statement

The raw data supporting the conclusions of this article will be made available by the authors, without undue reservation.

## Ethics statement

The animal study was reviewed and approved by The Fifth Affiliated Hospital of Zhengzhou University.

## Author contributions

SS: Data curation; Formal analysis; Investigation; Software; Validation; Visualization; Writing—original draft. FR: Formal analysis; Software; Investigation; Validation. HQ: Formal analysis; Software; Investigation; Validation. IB: Formal analysis; Writing—review & editing. JY: Funding acquisition. DG: Funding acquisition. ACO: Writing—review & editing. MJL: Writing—review & editing. PZ: Conceptualization; Project administration; Resources; Supervision. YM: Methodology; Project administration; Formal analysis; Writing—review & editing. All authors contributed to the article and approved the submitted version.
