# Supplementary material for: Lactobacillus acidophilus NCFM and Lactiplantibacillus plantarum Lp-115 inhibit Helicobacter pylori colonization and gastric inflammation in a murine model
Source: Front Cell Infect Microbiol. 2023 Aug 9;13:1196084. doi: 10.3389/fcimb.2023.1196084 (PMC10445763; doi:10.3389/fcimb.2023.1196084)
Supplement: Supplementary file 1 [file Image_1.pdf]

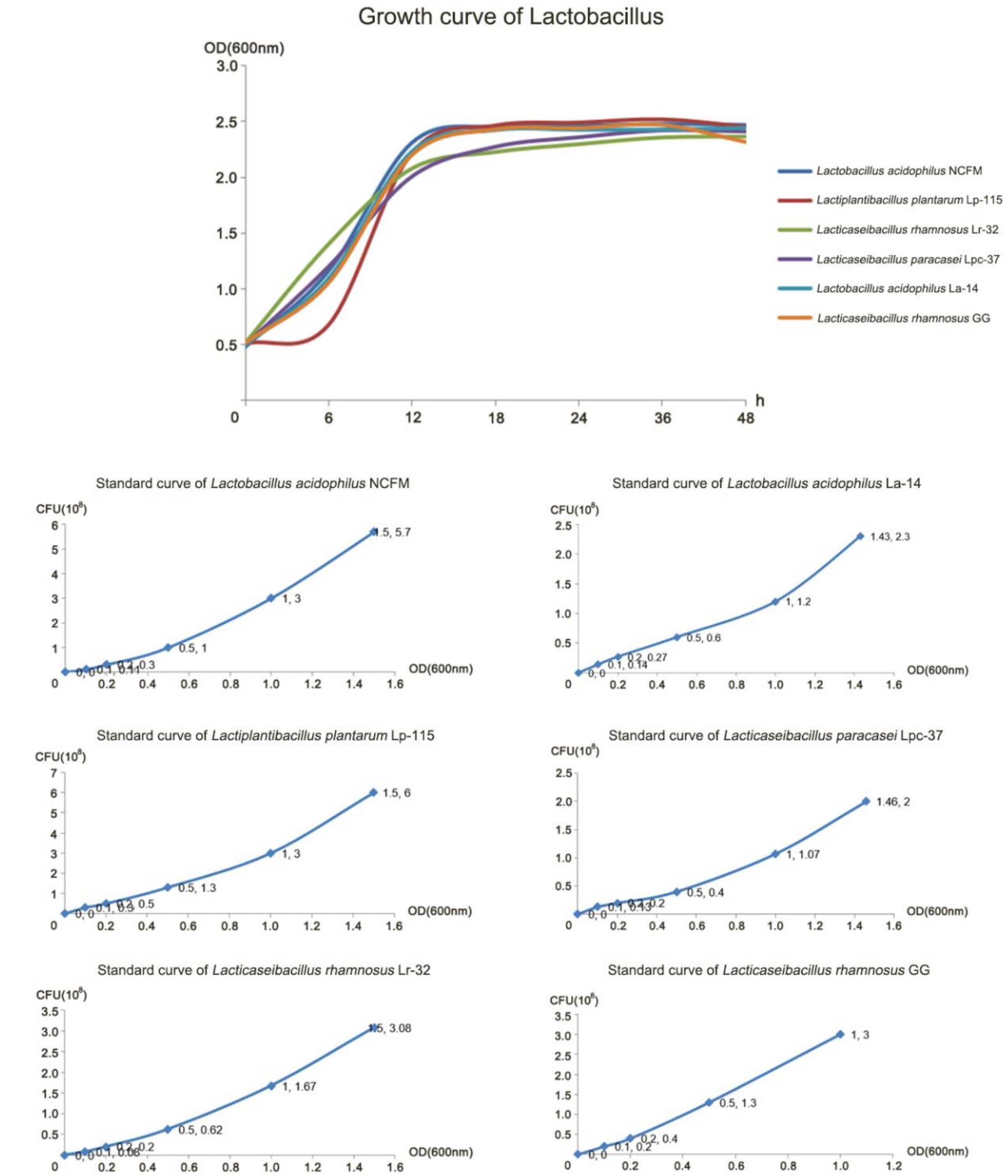

Figure S1. Growth curve and standard curve of each Lactobacillus.

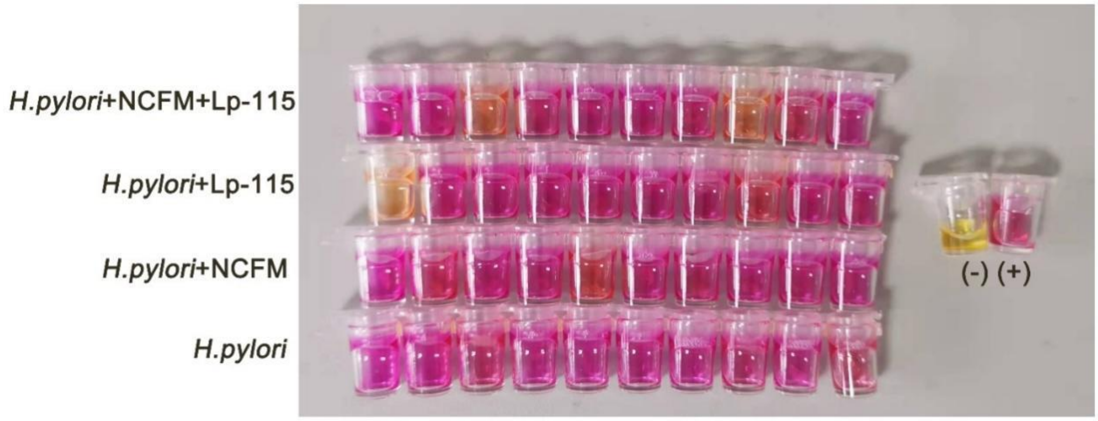

Figure S2. Rapid urease test assay for mice model. Two tubes on the right indicate control results: yellow is negative (-) and red or pink is positive (+). The groups in the left from top to bottom are *H. pylori* + *Lactobacillus acidophilus* NCFM + *Lactiplantibacillus plantarum* Lp-115 group, *H. pylori* + *L. plantarum* Lp-115 group, *H. pylori* + *L. acidophilus* NCFM group and *H. pylori* group. It can be seen that some samples in the columns of the lactobacilli intervention group tested negative, while all samples in the *H. pylori* group tested positive.
